# Divergent Androgen Receptor and Beta-Catenin Signaling in Prostate Cancer Cells

**DOI:** 10.1371/journal.pone.0141589

**Published:** 2015-10-28

**Authors:** Eugine Lee, Susan Ha, Susan K. Logan

**Affiliations:** 1 Department of Biochemistry and Molecular Pharmacology, New York University School of Medicine, New York, NY, United States of America; 2 Department of Urology New York University School of Medicine, New York, NY, United States of America; 3 Stem Cell Biology Program, New York University School of Medicine, New York, NY, United States of America; Innsbruck Medical University, AUSTRIA

## Abstract

Despite decades of effort to develop effective therapy and to identify promising new drugs, prostate cancer is lethal once it progresses to castration-resistant disease. Studies show mis-regulation of multiple pathways in castration-resistant prostate cancer (CRPC), reflecting the heterogeneity of the tumors and also hinting that targeting androgen receptor (AR) pathway alone might not be sufficient to treat CRPC. In this study, we present evidence that the Wnt/β-catenin pathway might be activated in prostate cancer cells after androgen-deprivation to promote androgen-independent growth, partly through enhanced interaction of β-catenin with TCF4. Androgen-independent prostate cancer cells were more prone to activate a Wnt-reporter, and inhibition of the Wnt/β-catenin pathway increased sensitivity of these cells to the second-generation antiandrogen, enzalutamide. Combined treatment of enzalutamide and Wnt/β-catenin inhibitor showed increased growth repression in both androgen-dependent and -independent prostate cancer cells, suggesting therapeutic potential for this approach.

## Introduction

Prostate cancer is the most common form of cancer in males [1]. Given the key role of androgen receptor (AR) signaling in disease progression, the current conventional approach to treat prostate cancer is androgen deprivation therapy often combined with antiandrogen treatment. Despite the initial tumor regression, aggressive disease progresses to castration-resistant prostate cancer (CRPC), for which treatment is the major challenge in the field. New drugs targeting the AR pathway such as the second-generation antiandrogen, enzalutamide [2] and abiraterone which blocks intratumoral production of androgen [3] have been FDA approved for the treatment of CRPC. Despite the promise of these and other therapeutics, they extend life by only 6–8 months [4, 5], indicating the need for a new approach to treat advanced disease.

Due to the complex signaling networks in advanced disease, inhibition of one pathway might cause unpredictable responses [6, 7]. In this context, it has been suggested that prostate tumors may also activate alternative signaling pathways to compensate for the consequences of AR inhibition [8, 9]. Interaction between the PI3K and AR pathways has been well studied and in fact reciprocal feedback between the two pathways in PTEN-deleted prostate cancer has been reported, indicating the importance of targeting both pathways in PTEN-deleted disease [10]. More recently, upregulation of glucocorticoid receptor (GR) was reported in enzalutamide-resistant tumors [11], and shown to be necessary for the resistant phenotype. Therefore, therapeutic approaches that concomitantly target multiple pathways might be more effective in treating CRPC [6].

Recent genome sequencing data has shown misregulation of the Wnt-pathway in prostate cancer with disease progression. The comparative analysis of two separate whole-exome sequencing sets of data, one from primary tumors [12] and the other from lethal castration-resistant metastatic tumors [13], revealed that the APC (adenomatous polyposis coli) gene was frequently mutated in primary tumors but more significantly mutated in advanced disease [14]. In fact, the later data shows that the Wnt-pathway is one of the most significantly mutated pathways in CRPC [13]. Consistent with this, WNT16B secretion in the tumor microenvironment promoted treatment-resistance in prostate cancer through activation of the Wnt/β-catenin pathway [15]. More recently it was reported that 18% of cases of metastatic castrate resistant prostate cancer exhibited alterations in Wnt pathway signaling [16]. These reports suggest that the Wnt/β-catenin pathway may be one of the compensatory pathways activated in prostate cancer in response to androgen deprivation therapy. Supporting this idea, the expression of an activating mutation of β-catenin in mouse prostate enabled continuous prostatic growth after castration [17].

Our group previously published proof of concept studies showing that a small molecule inhibitor of the Wnt/β-catenin pathway, iCRT3 (shortened to C3) could decrease AR mRNA expression and transcription of downstream target genes by interfering with β-catenin/TCF interaction on the AR promoter. We also showed that C3 could interfere with AR and β-catenin protein interaction. The later protein interaction studies were performed in the presence of high levels of androgen to stabilize AR protein levels so that they were not decreased in the presence of β-catenin inhibitor [18].

Work described in this manuscript shows that the activity of the Wnt/β-catenin pathway is low in prostate cancer cells likely due to the preference for β-catenin interaction with AR rather than TCF4 in these cells. We observe that suppression of AR activity by androgen-deprivation, antiandrogen treatment or AR knockdown promoted Wnt/β-catenin-target gene expression and this correlated with increased interaction between TCF4 and β-catenin. The enhanced activation of the Wnt/β-catenin pathway caused growth of androgen-dependent LNCaP cells in the absence of androgen or in the presence of antiandrogen. Activation of the Wnt/β-catenin pathway was also examined in an androgen-independent subline of LNCaP cells, LNCaP-abl (abl). Abl cells were generated by continuous passage in androgen depleted media and selected for their ability to proliferate in the androgen deprived condition [19]. Abl cells were more prone to Wnt/β-catenin activation than LNCaP cells, and inhibition of β-catenin activity by a small molecule inhibitor or siRNA increased enzalutamide sensitivity in abl cells. Furthermore, combined treatment of enzalutamide and a Wnt/β-catenin inhibitor exhibited increased growth inhibition in both LNCaP and abl cells, indicating the therapeutic potential of this approach.

## Materials and Methods

### Cell culture

LNCaP (ATCC, CRL-1740) and LNCaP-abl (abl) [19] (gift by Z. Culig) cells were cultured in RPMI 1640 (Cellgro) supplemented with 10% FBS (Hyclone), and 10% charcoal stripped FBS (CFBS; serum depleted of steroids including androgens), respectively, and 1% penicillin-streptomycin (Cellgro). 22Rv1 (ATCC, CRL-2505) and HEK293 (ATCC, CRL-1573) cells were cultured in DMEM (Cellgro) supplemented with 10% FBS and 1% penicillin-streptomycin. The following compounds were used to treat cells: C3 (ChemDiv, C523-1410), enzalutamide (Selleck Chemicals) and GSK-3 inhibitor (CHIR99021; Stemgent). For cell proliferation, qRT-PCR, immunoblot, immunoprecipitation and ChIP assays, LNCaP cells were hormone deprived in 5% CFBS media for 2–3 days and then treated with androgen with or without the above compounds.

### Stable integration of eGFP Wnt-reporter in prostate cancer cells

The lentiviral eGFP Wnt reporter, 7xTcf-eGFP/SV40-mCherry was purchased from Addgene (24304). Packaging cells (HEK293T/17) (ATCC, CRL-11268) were transiently transfected with 6 μg of eGFP Wnt reporter construct, 4 μg of packaging plasmid (psPAX2) and 2 μg of envelope expressing plasmid (pMD2.G) using Lipofectamine 2000 reagent (Invitrogen) according to manufacturer’s instructions. Media conditioned by the transfectants was collected after 24 and 48 h. LNCaP, abl and 22Rv1 cells were infected by incubation in conditioned media overnight and allowed to recover for a day. Infected cells were passaged and selected by mCherry expression using flow cytometry.

### Quantitative real-time RT-PCR (qRT-PCR)

Total RNA was isolated using the RNeasy kit (Qiagen), and then reverse transcribed at 55°C for 1 h using Superscript III reverse transcriptase and oligo- (dT) 20 primers (Invitrogen). Real-time PCR was performed using gene-specific primers and 2XSYBR green Taq-ready mix (Sigma). Data were analyzed by the DDCT method using RPL19 as a control gene and normalized to control samples, which were arbitrarily set to 1.

### Immunoblot, immunoprecipitation and immunostaining

For immunoblot analysis, cells were lysed in Triton buffer and supplemented with 1 mM PMSF, 1 mM Na_3_VO_4_, 10 mg/ml of leupeptin and 10 mg/ml of aprotinin. Protein lysates were subjected to SDS-PAGE and immunoblotted with the following antibodies against: AR (441), β-catenin (H-102), TCF4 (H-125, Santa Cruz Biotechnology); active β-catenin (clone 8E7, Millipore); tubulin (Covance). Protein bands were visualized using ECL Western Blotting detection reagents (GE Healthcare). Image J (NIH) software was used to quantitate protein levels.

In immunoprecipitation experiments cells were lysed as described above. Primary antibodies listed above were added to at least 1.5 mg of total protein and incubated overnight at 4°C followed by the addition of Protein A/G agarose beads (Santa Cruz Biotechnology) for 2 h. Immune complexes were extensively washed with Triton buffer and solubilized using Laemmli sample buffer (BioRad). Normal mouse IgG (Santa Cruz Biotechnology) or normal rabbit sera (Sigma) were used as controls.

For immunofluorescence staining, cells were fixed with 4% formaldehyde in PBS for 20 min, washed with PBS three times, permeabilized with 0.2% Triton-X in PBS for 20 min, blocked with 5% normal goat and 5% normal horse serum in PBS for 1 h, and incubated with antibody against active β-catenin (Millipore) diluted in blocking buffer, overnight at 4°C. The following day, cells were washed with PBS three times and then incubated with secondary antibodies conjugated with FITC (Vector Lab) at room temperature for 1 h. Subsequently, cells were washed in PBS three times and mounted with DAPI mounting solution (Vector Lab). To analyze eGFP and mCherry levels in cells stably integrated with 7xTcf-eGFP/SV40-mCherry constructs, cells were fixed, washed and permeabilized as described above and mounted with DAPI mounting solution.

### Cell proliferation assay

For the CyQUANT cell proliferation assay, 3 to 4 X 10^3^ cells were plated in each well of black 96 well plate. Cells were plated in hormone-deprived media containing 5% CFBS and cultured for 2 to 3 days, and then treated with indicated reagents for each experiment. The reagents were added every two days with more media and an equal amount of vehicle added to the control group. At 2–3 day intervals, CyQUANT assay (Invitrogen, C35006) was performed according to manufacturer’s instructions and analyzed on a SpectraMax M5 plate reader (Molecular Devices) running SoftMax Pro® software.

### Chromatin immunoprecipitation (ChIP) assay

ChIP was performed as previously described [20]. Proteins were double cross-linked with DSP (Pierce) for 20 min and 1% formalin for 10 min. Cells were lysed, nuclei collected and resuspended in sonication buffer, and sonicationed for 12 min (30 sec. on, 30 sec. off) in a Bioruptor sonicator (Diagenode, model XL). Sonicated lysates were pre-cleared for 2 h with Protein A/G agarose beads blocked with salmon sperm DNA (Millipore). Supernatants were then incubated overnight with the following antibodies: a mixture AR (441) and AR (N-20), or β-catenin (H-102). Control ChIP was performed with normal mouse IgG and normal rabbit IgG sera. Immunocomplexes were then washed and cross-linking reversed. DNA was isolated with the Qiagen PCR purification kit and qPCR was performed. Relative enrichment was calculated as a percentage of 4% input normalized to IgG.

### RNA-interference (RNA-i)

For β-catenin and AR knockdown, siGENOME SMARTpool siRNAs against β-catenin or AR were used (Dharmacon). For APC knockdown, the pool of three Silencer® Select siRNAs against APC (Ambion, s1433, s1434 and s1435) were used. Cells were transfected with HiPerFect transfection reagent (Qiagen) according to manufacturer’s instructions. 1 X 10^5^ cells were plated in each well of a 24 well plate in hormone-deprived media containing 5% CFBS and the mixture of HiPerFect reagent and siRNAs were added directly on top of the cells. Cells were cultured for 2 days and then treated with indicated reagents for each experiment. For CyQUANT proliferation assay, 3 to 4 X 10^3^ cells were plated in each well of black 96 well plates in hormone-deprived media containing 5% CFBS and the mixture of HiPerFect reagent and APC siRNAs were added directly on top of the cells. Cells were cultured for 2 days and then treated with indicated reagents for each experiment. CyQUANT assay was performed at 2–3 day intervals as described above.

The cell lines stably depleted of β-catenin were generated with lentiviral pGIPZ shRNA against β-catenin (Open Biosystems, RHS4430-98912789) or a control shRNA (Open Biosystems, RHS4743). After infection, cells were plated at a very low density and selected for 10 days with 1 μg/ml puromycin (Sigma). Each resistant cell colony was collected and expanded in the selection media to screen for beta-catenin protein levels. Two cell lines showing moderate reduction of β-catenin were selected to test enzalutamide sensitivity (sh-β-cat-1 and -2) to minimize the robust growth inhibitory effect of β-catenin knockdown (25).

## Results

### Androgen treatment represses Wnt-reporter activity in androgen-dependent LNCaP cells

**Fig 1:**
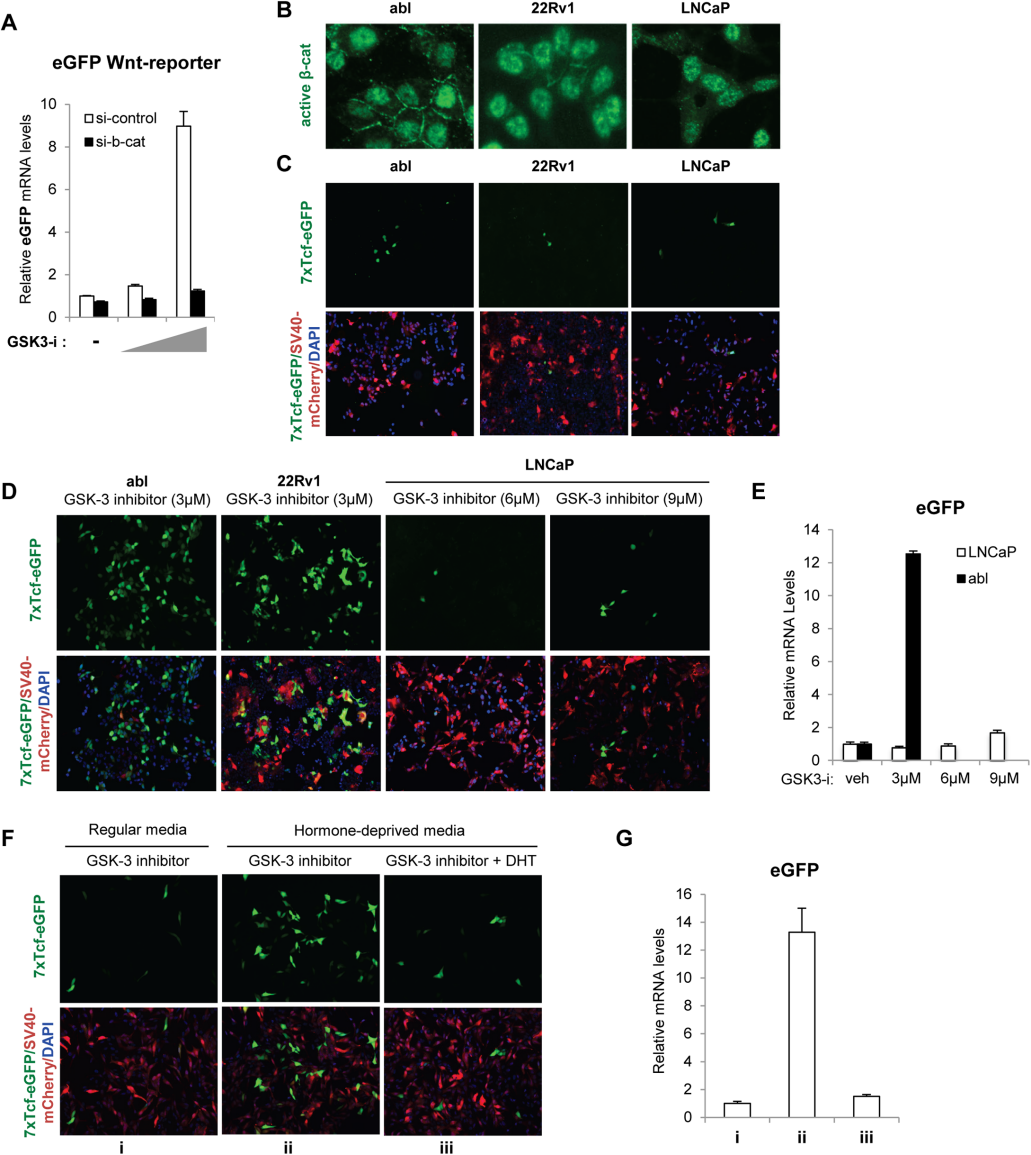
Prostate cancer cells show low Wnt-reporter activity and hormone-deprivation enhances activation of Wnt-reporter in LNCaP cells. *A*, -catenin knockdown diminished the Wnt-reporter activation in response to GSK-3 inhibitor. abl cells were infected with eGFP Wnt-reporter (7xTcf-eGFP/SV40-mCherry) and transfected with si-control or si--catenin. Cells were cultured for 2 days and then treated with vehicle, 2 μM or 3 μM GSK-3 inhibitor for 24 h. The relative mRNA levels of eGFP indicating Wnt activity were analyzed by qRT-PCR. *B*, The levels of nuclear -catenin (green) in LNCaP, abl and 22Rv1 prostate cancer cells were determined by immunofluorescence staining. *C*, Representative fluorescence images showing the low level of eGFP Wnt reporter activity in LNCaP, abl and 22Rv1 cells: Wnt activity (green), presence of cells (mCherry, red), and nuclear location (DAPI, blue). *D and E*, Androgen-independent abl and 22Rv1 cells are more prone to activation of Wnt-reporter. Cells were treated with GSK-3 inhibitor for 24 h and subjected to fluorescence imaging (*D*) or qRT-PCR to measure relative mRNA levels of eGFP (*E*). In *A-E*, experiments were performed in the normal growth media of each cell line as described in the Materials and Methods. *F and G*, Hormone-deprivation enhanced activation of Wnt-reporter in LNCaP cells, which is diminished by androgen treatment. Cells were treated with 9 μM GSK-3 inhibitor in complete (i) or hormone-deprived media with/without 10 nM di-hydrotestosterone (DHT) for 24 h (ii and iii). Cells were then subjected to fluorescence imaging (*F*), or qRT-PCR to measure relative mRNA levels of eGFP (*G*). The data presented is representative of three independent experiments and the indicated error is the standard deviation.

To study the Wnt/β-catenin pathway activity in prostate cancer, we monitored endogenous Wnt/β-catenin activity on a Wnt-reporter upstream of eGFP (7xTcf-eGFP/SV40-mCherry). The construct also expresses mCherry under the constitutively active SV40 promoter indicating positively infected cells [21]. LNCaP, LNCaP-abl (abl) and 22Rv1 prostate cancer cells were infected with lentivirus containing the reporter construct and cells stably integrated with the construct were selected by flow cytometry using mCherry expression. Wnt reporter gene activity was tested using GSK-3 inhibitor (GSK3-i; CHIR99021), a potent Wnt-activator [22, 23]. eGFP Wnt reporter activity was increased in the presence of GSK3-i, shown by the increased transcription of eGFP in LNCaP-abl cells stably expressing the reporter. The eGFP transcription was diminished in the presence of siRNA targeting β-catenin (Fig 1A). Using this reporter, we examined activity of the Wnt/β-catenin pathway in androgen-dependent LNCaP cells, and androgen-independent LNCaP-abl and 22Rv1 cells [24, 25]. In spite of abundant levels of active, nuclear β-catenin (Fig 1B), the three cell lines showed low basal activity of Wnt-reporter (Fig 1C). Treatment with GSK-3 inhibitor (3μM) activated Wnt-reporter activity in androgen-independent abl and 22Rv1 cells (Fig 1D), suggesting that increased levels of β-catenin were required to activate Wnt/β-catenin-responsive transcription. However, treatment of androgen-dependent LNCaP cells with 3μM GSK3-i had very little effect on the Wnt reporter (data not shown). Additionally, the Wnt-reporter was not activated in the presence of higher concentrations of GSK-3 inhibitor (6 or 9μM) (Fig 1D). Fig 1E shows the relative mRNA levels of eGFP in each condition, with increased eGFP transcription in GSK-3 inhibitor treated abl cells, but not in LNCaP cells.

Given that β-catenin interacts with AR in an androgen-dependent manner [26–28], we reasoned that androgen-deprivation might enhance β-catenin interaction with TCF, thereby increasing Wnt-reporter activity. In support of this idea, treatment of LNCaP cells with GSK-3 inhibitor in regular hormone containing media (condition i) compared to the hormone-deprived media (condition ii) resulted in increased reporter activation (Fig 1F) in the hormone deprived condition. Treatment with di-hydrotestosterone (DHT) diminished this reporter activation (Fig 1F, condition iii), suggesting that androgen treatment represses Wnt reporter activity in LNCaP cells. The relative level of eGFP mRNA in each condition is shown in Fig 1G.

### Inhibition of AR activity enhances Wnt/β-catenin-responsive transcription

**Fig 2:**
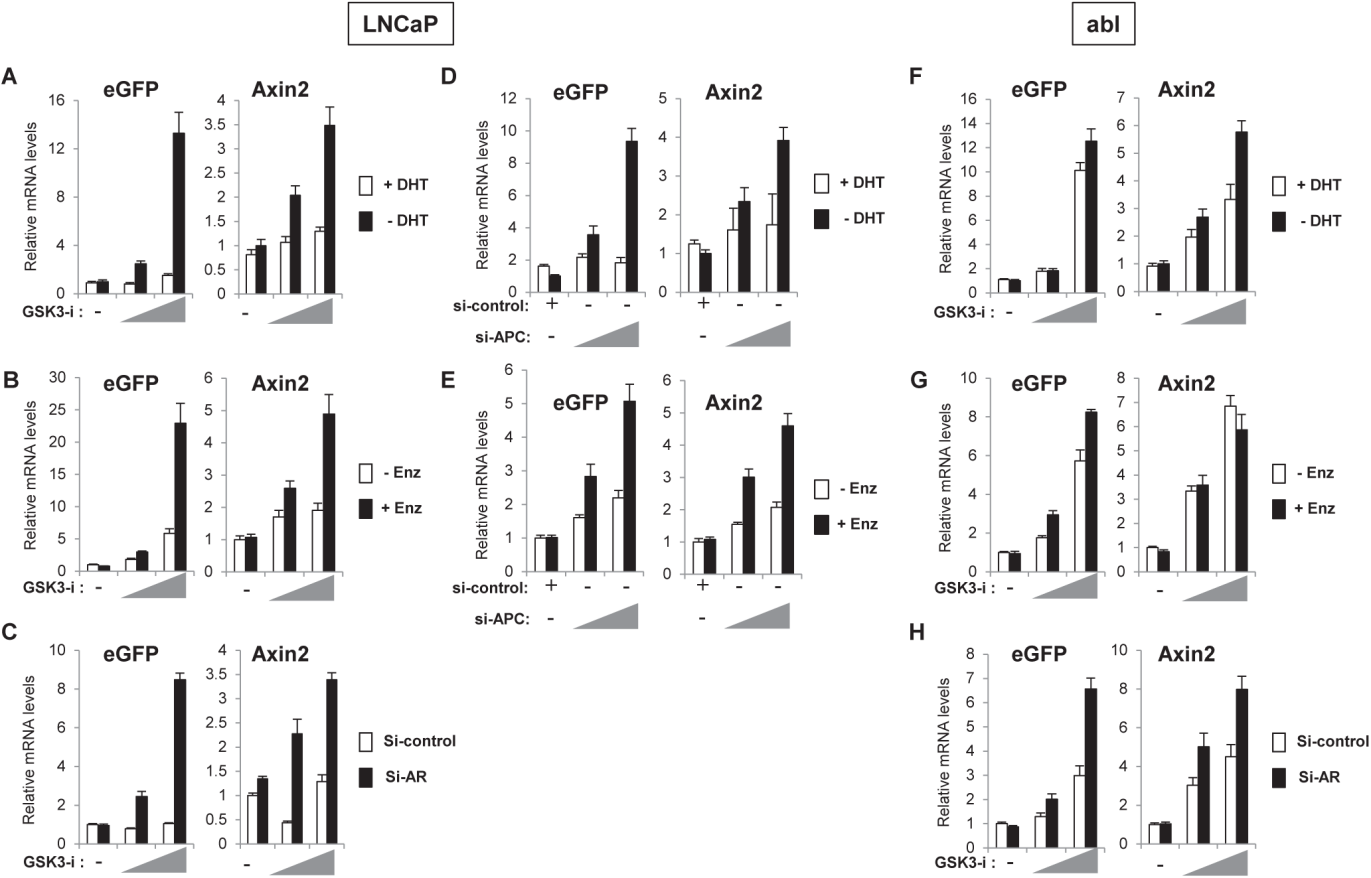
Inhibition of AR activity enhances Wnt/β-catenin target gene expression in response to GSK-3 inhibitor or APC knockdown. LNCaP cells were treated with 6 μM or 9 μM of GSK-3 inhibitor (*A-C*); abl cells were treated with and 2 μM or 3 μM of GSK-3 inhibitor (*F-H*). *A and F*, Cells were hormone-deprived for 3 days and then treated with vehicle or increasing concentration of GSK-3 inhibitor with/without 10 nM DHT for 24 h. *B and G*, Cells were treated with vehicle or increasing concentration of GSK-3 inhibitor with/without 10 μM enzalutamide (Enz) for 24 h. *C and H*, Cells were transfected with si-control or si-AR, cultured for 2 days and then treated with vehicle or increasing concentration of GSK-3 inhibitor for 24 h. *D and E*, LNCaP cells were transfected with si-control or increasing amounts of si-APC and either hormone-deprived for 2 days and then treated with vehicle or 10 nM DHT for 24 h (*D*), or cultured in complete media for 2 days and then treated with vehicle or 10 μM enzalutamide for 24 h (*E*). The relative mRNA levels of indicated genes are analyzed by qRT-PCR. The data presented is representative of three independent experiments and the indicated error is the standard deviation.

As we observed increased activation of the Wnt-reporter in hormone-deprived conditions, we hypothesized that inhibition of AR activity might enhance Wnt/β-catenin-responsive transcription. To test this idea, AR activity was repressed by either hormone-deprivation, antiandrogen treatment (enzalutamide; [2]) or siRNA-mediated AR depletion. Cells were treated with increasing concentrations of GSK-3 inhibitor to induce Wnt/β-catenin-responsive transcription and the relative mRNA levels of the Wnt-reporter (eGFP) and endogenous Wnt/β-catenin target gene, *Axin2*, were analyzed. Compared to control cells, higher induction of Wnt-reporter and *Axin2* mRNA levels were observed in LNCaP cells cultured in hormone-deprived media, in the presence of enzalutamide or treated with AR siRNA (Fig 2A–2C). As an alternative to GSK-3 inhibitor treatment we also repeated the experiment using APC knockdown to activate the Wnt/β-catenin pathway. APC is a component of the β-catenin destruction complex and APC deletion or loss-of-function mutation has been shown to stabilize β-catenin and activate Wnt/β-catenin-responsive transcription [29, 30]. Consistent with results in GSK-3 inhibitor treated cells (Fig 2A–2C), APC knockdown induced higher activation of the Wnt-reporter gene and *Axin2* transcription in hormone-deprived or enzalutamide treated cells (Fig 2D and 2E), suggesting that AR repression promotes Wnt/β-catenin-activation in LNCaP cells. However, abl cells exhibited high levels of Wnt reporter and *Axin2* expression in response to GSK-3 inhibitor that were only modestly increased in the absence of DHT or presence of enzalutamide (Fig 2F and 2G) with the exception of *Axin2*, which was unaffected by enzalutamide treatment compared to control cells (Fig 2G). Depletion of AR by siRNA showed an increase in Wnt reporter gene activity and *Axin2* mRNA in both LNCaP and abl cells (Fig 2C and 2H) suggesting that depletion of AR protein results in release of more β-catenin into a cellular pool available to activate the Wnt/β-catenin pathway.

Altogether, these results suggest that in androgen-dependent LNCaP cells, inhibition of AR may allow the tumor cells to activate the Wnt/β-catenin pathway. Under conditions of hormone deprivation this may occur upon exposure to Wnt-signals likely present in the tumor microenvironment [15, 31, 32]. In contrast, androgen-independent abl cells that are derived from LNCaP cells, appear poised for Wnt/β-catenin-activation in both presence and absence of AR activity, indicating that the interaction between AR and Wnt/β-catenin pathways may have been modified during the progression to androgen independence.

### Hormone deprivation enhances β-catenin interaction with TCF4

**Fig 3:**
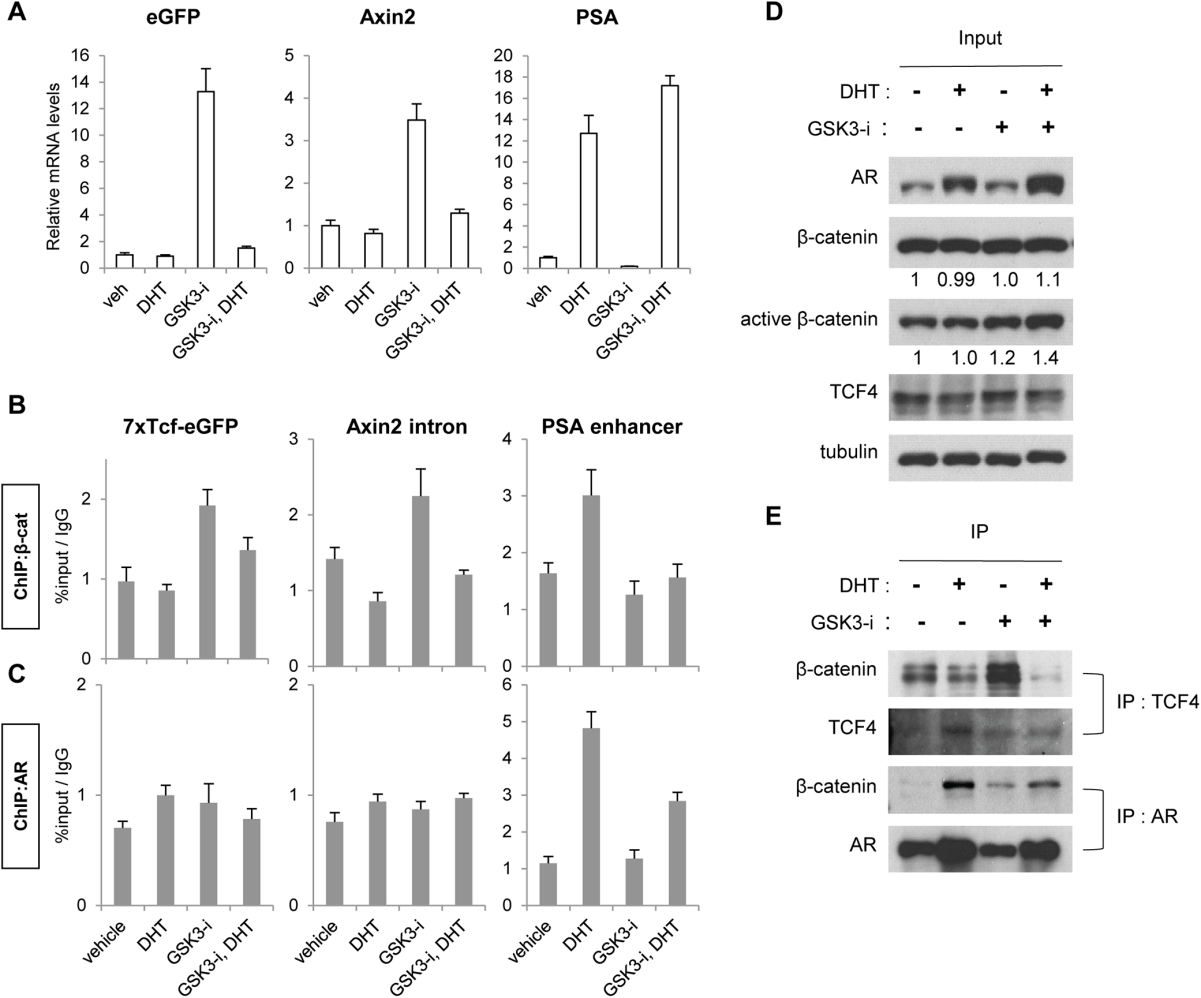
Hormone deprivation increased β-catenin interaction with TCF4 and β-catenin binding to TCF binding sites. *A*, Expression of Wnt/-catenin target genes (eGFP reporter and *Axin2*) and the AR target gene (*PSA*) was analyzed. LNCaP cells were hormone-deprived for 3 days and then treated with vehicle, 10 nM DHT, 9 μM GSK-3 inhibitor or a combination of both for 24 h. *B and C*, -catenin or AR binding on target genes was analyzed using chromatin-immunoprecipitation. LNCaP cells were hormone-deprived for 3 days and then treated with vehicle, 100 nM DHT, 9 μM GSK3-i or a combination of both for 4 h. *D and E*, -catenin interaction with TCF4 or AR was analyzed using co-immunoprecipitation. LNCaP cells were hormone-deprived for 3 days and then treated with vehicle, 100 nM DHT, 9 μM GSK-3 inhibitor or a combination of both for 4 h. The protein lysates were either immunoblotted with indicated antibodies (*D*) or subjected to co-IP studies (*E*) Quantification of β-catenin and active β-catenin protein levels are shown below panels in (*D*). Relative densitometry is normalized to vehicle alone, set to 1. The data presented is representative of three independent experiments and the indicated error is the standard deviation.

Given that β-catenin interacts with AR or TCF to activate AR or Wnt/β-catenin-responsive transcription, respectively, in prostate cancer [33, 34], we examined the β-catenin occupancy on AR and TCF binding sites using chromatin immunoprecipitation (ChIP) assays. LNCaP cells were treated with DHT, GSK-3 inhibitor or a combination of both for 4 h in hormone-deprived media. The relative mRNA levels of Wnt/β-catenin (eGFP Wnt-reporter and *Axin2*) and AR-target (*PSA*) genes in each condition were analyzed (Fig 3A). β-catenin occupancy on TCF or AR binding sites was also examined (Fig 3B) to determine if β-catenin recruitment correlated with target gene expression. As expected, Wnt-reporter and *Axin2* mRNA levels were increased in GSK-3 inhibitor treated cells, but diminished in the presence of DHT and GSK-3 inhibitor (Fig 3A). Consistent with the mRNA levels, β-catenin was recruited to TCF binding sites on the Wnt-reporter and *Axin2* in response to GSK-3 inhibitor, but not in the cells co-treated with GSK-3 inhibitor and DHT (Fig 3B). Transcription of *PSA* occurred in response to DHT, and this was unaffected by co-treatment with DHT and GSK-3 inhibitor (Fig 3A). β-catenin occupancy was observed at the *PSA* enhancer upon DHT treatment, but was diminished upon co-treatment with DHT and GSK-3 inhibitor (Fig 3B), suggesting that in this context β-catenin is not essential for *PSA* transcription. AR was also analyzed; showing occupancy on *PSA* in response to DHT treatment but no binding was detected on TCF sites of Wnt/β-catenin target genes in any treatment (Fig 3C).

To determine if AR and β-catenin occupancy on target genes correlates with β-catenin binding to either AR or TCF4 protein, we conducted co-immunoprecipitation assays. LNCaP cells were treated as described for the ChIP assays above, and protein lysates were immunoprecipitated with β-catenin antibody. Fig 3D shows that AR is stabilized by DHT treatment. While total levels of β-catenin were unchanged by 4 hours of GSK-3 inhibitor treatment, GSK-3 inhibition slightly increased the protein expression of the active form of β-catenin (unphosphorylated at serine 37 and threonine 41, [35]), independent of the presence of DHT (Fig 3D). Overall, DHT treatment increased AR/β-catenin interaction and decreased TCF4/β-catenin interaction compared to vehicle treatment (Fig 3E), as reported previously [34, 36]. Consistent with the ChIP results (Fig 3B), GSK-3 inhibitor enhanced the TCF4/β-catenin interaction but combinatory treatment with DHT diminished this interaction (Fig 3E). In addition, cells treated with DHT showed enhanced AR/β-catenin interaction, which was decreased in the presence of GSK-3 inhibitor (Fig 3E) consistent with decreased AR on the on the *PSA* enhancer in the presence of GSK-3 inhibitor and DHT (Fig 3C). It is unknown why AR binding to *PSA* and interaction with β-catenin are decreased when DHT is co-treated with GSK-3 inhibitor, but this reduced level of AR on the *PSA* enhancer was still sufficient for *PSA* transcription (Fig 3A, PSA mRNA levels in DHT vs DHT+GSK3-i).

In summary, both ChIP and co-IP assay results suggest that β-catenin interaction with TCF4 (Fig 3E) and the recruitment to Wnt/β-catenin target genes (Fig 3B) was increased in the absence of androgen, consistent with the enhanced Wnt/β-catenin-responsive transcription under hormone-depleted conditions (Figs 1F, 2A and 2D).

### Inhibition of AR and Wnt/β-catenin pathways increases growth repression of LNCaP cells

**Fig 4:**
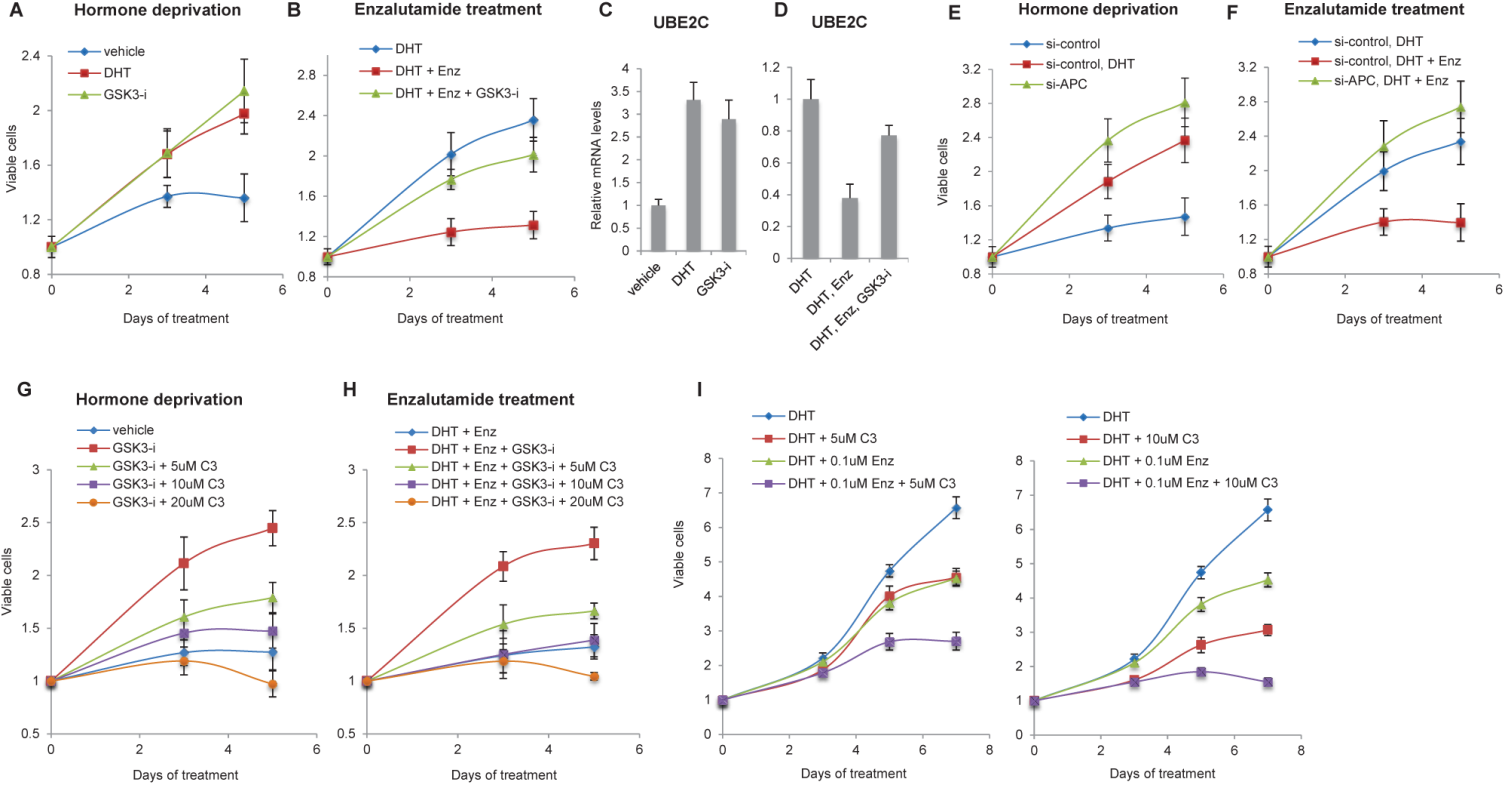
Wnt/β-catenin activation promotes androgen-independent growth of LNCaP cells and inhibition of both AR and Wnt/β-catenin pathways shows increased growth inhibition of LNCaP cells. *A-F*, GSK-3 inhibitor treatment or APC knockdown promotes growth of LNCaP cells in hormone-deprived or enzalutamide (Enz) treated media. *A and B*, Cells were hormone-deprived for 3 days and then treated with vehicle, 10 nM DHT or 6 μM GSK3-i (*A*), or 10 nM DHT with/without 10 μM Enz or 6 μM GSK3-i (*B*) every two days. *C and D*, Cells were treated as described in (*A*) or (*B*) for 24 h and then the relative mRNA levels of *UBE2C* were analyzed by qRT-PCR. *E and F*, Cells were transfected with si-control or si-APC, hormone-deprived for 2 days and then treated with vehicle or 10 nM DHT (*E*), or 10 nM DHT with/without 10 μM Enz (*F*) every two days. *G and H*, Treatment of cells with Wnt/β-catenin inhibitor (C3) diminishes the growth-promoting effect of GSK3-i. LNCaP cells were hormone-deprived for 3 days and then treated with vehicle or 6 μM GSK3-i (*G*), or 10 nM DHT plus 10 μM Enz (*H*) with/without increasing concentrations of C3 every two days. *I*, Co-treatment of Enz and C3 shows increased growth inhibition of LNCaP cells. Cells were hormone-deprived for 3 days and then treated with 10 nM DHT with/without 5 μM or 10 μM C3, 0.1 μM Enz or a combination of both compounds every two days. Cell viability is represented by relative fluorescence units normalized to time zero (prior to treatments). The data presented is representative of three independent experiments and the indicated error is the standard deviation.

Since one of the cellular responses of the Wnt-pathway is to promote proliferation [37, 38], we tested if the enhanced activation of Wnt/β-catenin pathway in the absence of androgen or presence of enzalutamide (Fig 2A, 2B, 2D and 2E) also promoted cell proliferation. LNCaP cells cultured in hormone-deprived media were growth arrested as expected [39] but GSK-3 inhibitor treatment or APC knockdown induced growth similar to DHT treatment (Fig 4A and 4E). Enzalutamide treatment also repressed growth of LNCaP cells as previously observed [2] however this growth inhibitory effect was relieved upon GSK-3 inhibitor treatment or APC knockdown (Fig 4B and 4F). We also examined transcription of an M phase cell cycle regulatory gene, *UBE2C*, previously shown to be important for growth of androgen-independent prostate cancer cells [40]. The GSK-3 inhibitor treatment resulted in upregulation of *UBE2C* transcription similar to the DHT treatment (Fig 4C), as well as de-repression of *UBE2C* in enzalutamide co-treated cells (Fig 4D). These results suggest that Wnt/β-catenin activation promotes growth of LNCaP cells in the absence of androgen or in the presence of enzalutamide, accompanied by upregulation of *UBE2C*. Treatment of cells with an inhibitor of the Wnt/β-catenin pathway, iCRT3 (C3) [41], diminished the growth-promoting effect of GSK-3 inhibitor in a dose-responsive manner (Fig 4G and 4H), further indicating that the Wnt/β-catenin pathway directs androgen-independent growth of LNCaP cells.

Since inhibition of AR activity resulted in enhanced Wnt/β-catenin-responsive transcription (Fig 2) and cell growth (Fig 4A, 4B, 4E and 4F), we hypothesized that simultaneous inhibition of both AR and Wnt/β-catenin pathways might be more effective than targeting one pathway alone. To test this idea, LNCaP cells were treated with either vehicle, enzalutamide or C3, or a combination of the two compounds. We used a sub-optimal concentration of the compounds (0.1μM of enzalutamide and 5μM or 10μM of C3) to determine the difference between single treatment versus co-treatment. Fig 4I shows that the combined treatment of enzalutamide and C3 has increased effects on growth inhibition of LNCaP cells, indicating the therapeutic potential of targeting both AR and Wnt/β-catenin pathways in prostate cancer.

### Inhibition of the Wnt/β-catenin pathway sensitizes abl cells to enzalutamide

**Fig 5:**
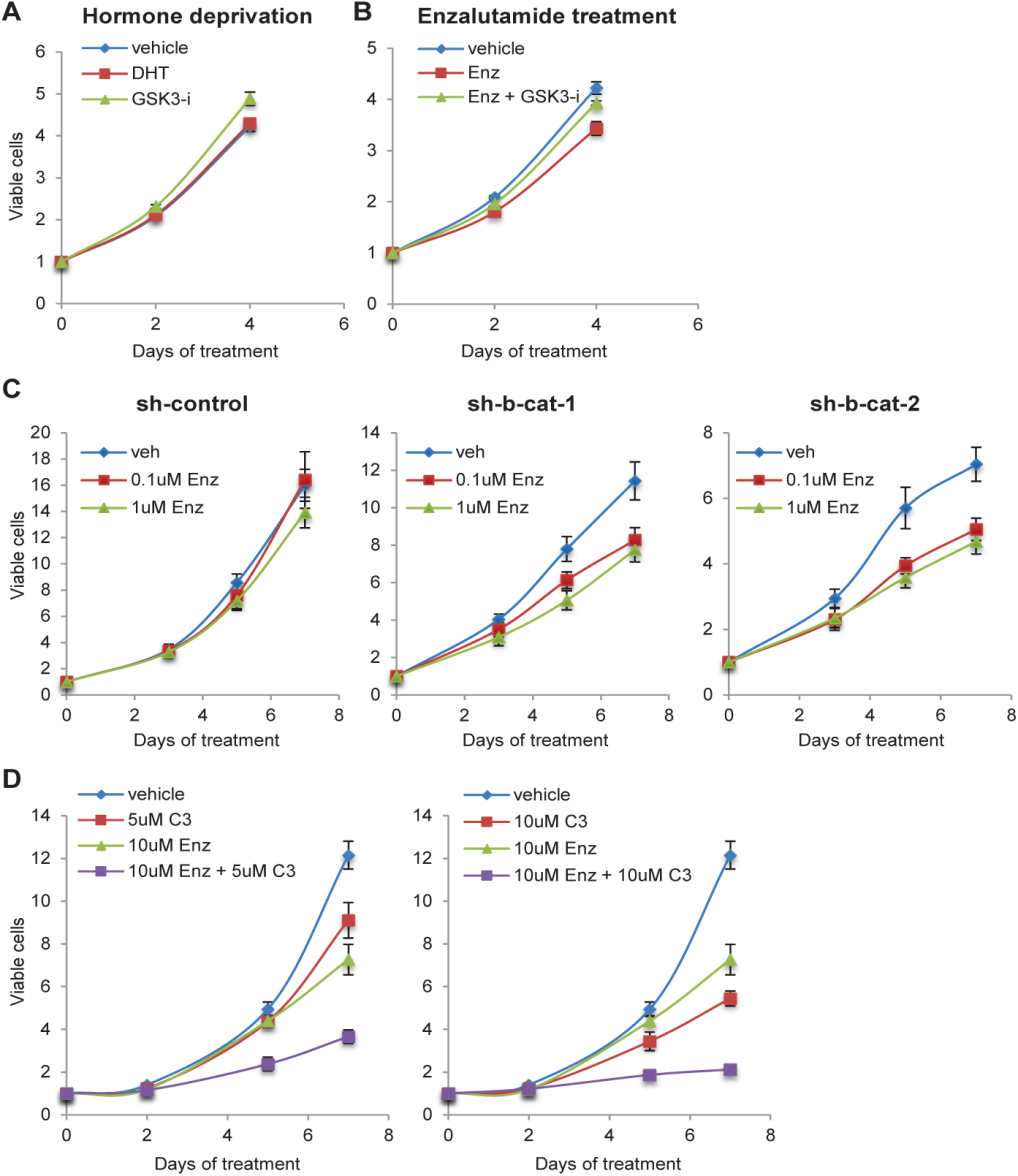
Inhibition of both AR and Wnt/β-catenin pathways shows increased growth inhibition of abl cells. *A and B*, Wnt/β-catenin activation shows no effect on growth of abl cells. Cells were treated with vehicle, 10 nM DHT or 6 μM GSK-3 inhibitor (GSK3-i) (*A*), or vehicle or 10 μM enzalutamide (Enz) with/without 6 μM GSK3-i (*B*) every two days. *C*, Inhibition of the Wnt/β-catenin pathway through β-catenin knockdown sensitized abl cells to Enz. Cells were stably infected with lentiviral vectors encoding control shRNA (sh-control) or -catenin shRNA (sh--cat-1 and 2) and cultured with treatment of vehicle, 0.1 μM or 1 μM Enz every two days. *D*, Co-treatment of Enz and Wnt/β-catenin inhibitor (C3) shows increased growth inhibition of abl cells. Cells were treated with vehicle, 5 μM or 10 μM C3, 10 μM Enz or a combination of both compounds every two days. Cell viability is represented by relative fluorescence units normalized to time zero (prior to treatments). The data presented is representative of three independent experiments and the indicated error is the standard deviation.

Contrary to what we observed in LNCaP cells, treatment of abl cells with GSK-3 inhibitor showed no effect on proliferation in either hormone-deprived or enzalutamide treated culture conditions (Fig 5A and 5B). In fact, consistent with their androgen-independent status, the growth of abl cells was not repressed by either hormone deprivation or enzalutamide treatment (Fig 5A and 5B). Our data suggests that abl cells are more prone to activate the Wnt/β-catenin pathway than LNCaP cells (Figs 1D, 1E and 2F–2H). Also, given that previous studies [17, 30] and our observations (Fig 4A and 4B) indicate that the Wnt/β-catenin pathway may promote androgen-independent growth of prostate cancer, we hypothesized that inhibition of Wnt/β-catenin pathway might sensitize abl cells to enzalutamide treatment. To test this idea, we examined the effect of β-catenin knockdown on enzalutamide treated abl cells. The β-catenin knockdown slowed the proliferation of abl cells as previously shown [18] (Fig 5C; vehicle in sh-control vs. sh-b-cat-1 and 2). While enzalutamide treatment did not affect growth of control cells (Fig 5C, sh-control), 0.1 μM of enzalutamide showed growth inhibition in β-catenin depleted cells (Fig 5C, sh-b-cat-1 and 2). Interestingly, 0.1 μM of enzalutamide treatment showed inhibition comparable to 1 μM (Fig 5C, sh-b-cat-1 and 2), suggesting that a lower concentration of AR antagonist may be effective under conditions of Wnt/β-catenin pathway inhibition. Next, we tested the effect of small molecule inhibition of the Wnt/β-catenin pathway in enzalutamide treated abl cells. The cells were treated with vehicle, enzalutamide, C3, or enzalutamide plus C3. Fig 5D shows a dose-responsive, additive effect of C3 treatment with enzalutamide, indicating that targeting the Wnt/β-catenin pathway can be a promising approach for enzalutamide resistant CRPC.

## Discussion

Extensive effort has been spent to develop more effective next-generation AR therapies to treat CRPC, including enzalutamide [2] and abiraterone [3]. However, resistance to these treatments inevitably occurs [42], hinting that targeting the AR pathway might not be sufficient, especially given the idea that increased crosstalk between distinct signaling pathways causes activation of AR target genes and regulatory networks in advanced prostate cancer [8, 9]. Similarly, despite the clinical success of targeted therapy in many cancer types, overcoming innate or acquired resistance remains a challenge [6, 43]. The emerging mechanism underlying acquired treatment resistance is that the growth-promoting signals are still active in cancer cells through activation of alternative signaling pathways. For example, kinase inhibitor treated cancers such as melanoma, lung, breast and colorectal cancers exhibit upregulation of other kinases and/or upregulation of ligands [44–46]. Thus, understanding the mechanisms of acquired resistance and development of combination therapy targeting the resistant pathways will be an effective approach to treat advanced cancers [6].

The crosstalk between AR and Wnt/β-catenin pathways in prostate cancer has been well studied [47, 48]. In addition, the major downstream effector of the canonical Wnt-pathway, β-catenin, was shown to be an AR co-activator [26, 28, 36]. More importantly, the AR gene is transcriptionally regulated by β-catenin through multiple TCF binding sites within the AR promoter [49]. Growing evidence has shown the increased mis-regulation of Wnt/β-catenin pathway in CRPC [13, 15]. However, the possibility that inhibition of the AR pathway engenders prostate cancer cells that are reliant on Wnt/β-catenin signaling has not been tested experimentally.

Here we showed that the Wnt/β-catenin pathway is repressed by AR in androgen-dependent LNCaP cells. We also showed that hormone-deprivation or antiandrogen treatment led to Wnt/β-catenin driven cellular proliferation (Fig 4A, 4B, 4E and 4F). Simultaneous inhibition of AR and Wnt/β-catenin pathways showed an increased effect on growth repression in LNCaP cells (Fig 4I). In addition, we observed that androgen-independent abl cells were more prone to activate Wnt/β-catenin responsive transcription (Figs 1D, 1E and 2F–2H), indicating a likely role of this pathway in disease progression. Furthermore, inhibition of the Wnt/β-catenin pathway using siRNA against β-catenin or a small molecule β-catenin inhibitor re-established the sensitivity to enzalutamide in abl cells (Fig 5C and 5D), suggesting that inhibition of the Wnt/β-catenin pathway can be an effective therapeutic approach to treat enzalutamide-resistant CRPC.

Previous studies indicate that AR and TCF4 have overlapping interaction domains on β-catenin [36, 50] and the competitive binding of AR and TCF4 to β-catenin has been shown in both prostate and colon cancer cells [34, 36]. The studies showed that androgen enhanced β-catenin interaction with AR and increased AR transcriptional activity, but resulted in repression of TCF/β-catenin responsive transcription [33, 34]. However, these earlier studies utilized the ectopic expression of a constitutively active β-catenin mutant, raising the concerns of non-specific effects. Furthermore, the status of the Wnt/β-catenin pathway has not been previously addressed in the absence of androgen (similar to conditions of androgen ablation therapy), to test if inhibition of AR activity enhances β-catenin interaction with TCF4.

Here, we activated endogenous β-catenin through inhibition of the β-catenin destruction complex via GSK-3 inhibition. We used GSK-3 inhibitor or siRNA against APC to show that the activation of Wnt/β-catenin responsive transcription was enhanced when AR activity was repressed (Fig 2). This was accompanied by increased β-catenin interaction with TCF4 (Fig 3E) and β-catenin recruitment to TCF binding sites on Wnt/β-catenin target genes (Fig 3B). Increased Wnt/β-catenin responsive transcription also promoted the cell growth in the absence of androgen (Fig 4A and 4E) or upon enzalutamide treatment (Fig 4B and 4F), indicating a likely role of the Wnt/β-catenin pathway in CRPC progression. In contrast, the Wnt/β-catenin pathway was readily activated in androgen-independent abl cells in both the presence and absence of androgen or enzalutamide (Fig 2F and 2G), and this activation did not further promote the abl cell growth (Fig 5A and 5B). These findings suggest that the crosstalk between AR and Wnt/β-catenin pathways might have been modified during development of androgen independence, consistent with previous evidence showing enhanced Wnt/β-catenin signaling in CRPC [33, 51–53]. Importantly, siRNA mediated or small molecule inhibition of Wnt/β-catenin pathway sensitized abl cells to enzalutamide treatment (Fig 5C and 5D), indicating that enhanced Wnt/β-catenin signaling might be one of the mechanisms that confer enzalutamide resistance.

One promising strategy to overcome acquired resistance is a rationally designed combination therapy against the resistant pathways based on pre-clinical studies [6]. Combination therapy has been proven to be effective in multiple cancer types, such as PI3K and MEK inhibition in KRAS or EGFR driven cancers [46] and mTOR and aromatase inhibition in breast cancer [54]. In prostate cancer, combined treatment with AR inhibitors and chemotherapeutic agents has been tested in the clinic, but the efficacy of targeting multiple pathways mis-regulated in advanced prostate cancer has not been well studied [42]. In this context, the Wnt/β-catenin pathway holds great potential to be tested in combination therapy with AR pathway inhibitors based on the well-known crosstalk between the AR and Wnt/β-catenin pathways [47, 48]. Supporting this idea, our data showed that the simultaneous inhibition of AR and Wnt/β-catenin pathways exhibited increased effects on growth repression of both LNCaP and abl cells (Figs 4I and 5D). Further studies such as testing the efficacy of this approach in vivo will be required.

In summary, this study showed that inhibition of AR activity caused increased activation of Wnt/β-catenin pathway and this increased activation promoted androgen-independent growth of prostate cancer cells. Simultaneous inhibition of AR and Wnt/β-catenin pathways showed increased growth repression of LNCaP and enzalutamide-resistant abl cells, indicating the therapeutic potential of targeting both pathways. The development of Wnt/β-catenin-inhibitors optimized for in vivo use will facilitate this approach.

## Abbreviations

AR: androgen receptor
APC: adenomatous polyposis coli
CRPC: castration-resistant prostate cancer
DHT: dihydrotestosterone
GSK3: glycogen synthase kinase 3
iCRT3: inhibitor of catenin responsive transcription 3
PI3K: phosphatidylinostitol-3 kinase
PTEN: phosphatase and tensin homolog
TCF4: Transcription factor 4
UBE2C: Ubiquitin-conjugating enzyme E2C
